# Adrenal insufficiency in patients with acquired immunodeficiency syndrome-an underestimated problem

**DOI:** 10.1186/1742-4690-9-S1-P143

**Published:** 2012-05-25

**Authors:** Deepak R Madi, Shashidhar Khanapure, John Ramapuram, Basavaprabhu Achappa, Sathish Rao

**Affiliations:** 1 Manipal University, Mangalore, India

## Introduction

Adrenal insufficiency is a serious complication of AIDS. Usually the integrity of HPA (Hypothalamo pituitary) axis is assessed by measuring cortisol response to 250µg of ACTH. Recent studies have shown that lower ACTH dose increases the sensitivity of the procedure. In the present study we have tried to estimate prevelance of adrenal insufficiency using low dose ACTH test (1µg).

## Primary objective

To estimate the prevelance of adrenal insufficiency in AIDS patients using low dose ACTH test.

## Research design and methods

50 patients with confirmed diagnosis of HIV were included in the study. History and physical examination were recorded. Lab investigations included Cd4 count, serum cortisol and low dose acth stimulation test. Data of patients with adrenal insufficiency (GROUP 1) was compared with those without adrenal dysfunction (GROUP 2). Stastical analysis was done using appropriate tests.

## Results

37/50 (74%) of study subjects had adrenal insufficiency. Basal cortisol in (GROUP 1) and (GROUP 2) was 10.09µg/dl and 21.95µg/dl (P < .05). Cortisol post Acth stimulation test in (GROUP 1) and (GROUP 2) was 9.49 µg/dl and 19.93µg/dl (P < .05). Mean Cd4 count in (GROUP 1) and (GROUP 2) was 138.7±56.17 cells/µl and 171.8.7±25.41cells/µl (P < .05). Blood glucose, serum sodium was low and serum potassium, eosinophil counts were high in (GROUP 1) when compared to (GROUP2).

## Conclusion

Adrenal insufficiency in patients with acquired immunodeficiency syndrome is a common problem in clinical practice.

